# Carbon Emissions From Patient Travel for Health Care

**DOI:** 10.1001/jamanetworkopen.2025.2513

**Published:** 2025-03-31

**Authors:** Hanna Zurl, Zhiyu Qian, Daniel R. Stelzl, Filippo Dagnino, Stephan M. Korn, Muhieddine Labban, Stuart R. Lipsitz, Marianne Leitsmann, Sascha Ahyai, Chad Ellimoottil, Stacy Loeb, Hari S. Iyer, Quoc-Dien Trinh, Alexander P. Cole

**Affiliations:** 1 Department of Urology, Medical University of Graz, Graz, Austria; 2 Department of Urology, Brigham and Women’s Hospital, Harvard Medical School, Boston, Massachusetts; 3 Center for Surgery and Public Health, Brigham and Women’s Hospital, Harvard Medical School, Boston, Massachusetts; 4 Department of Urology, University Medical Center Hamburg-Eppendorf, Hamburg, Germany; 5 Department of Urology, Humanitas Research Hospital–IRCCS, Milan, Italy; 6 Department of Urology, Medical University of Vienna, Vienna, Austria; 7 Department of Urology, University of Michigan, Ann Arbor; 8 Department of Urology and Population Health, New York University Langone Health, New York; 9 Section of Cancer Epidemiology and Health Outcomes, Rutgers Cancer Institute, New Brunswick, New Jersey

## Abstract

**Importance:**

The US health care sector accounts for about 8.5% of national greenhouse gas (GHG) emissions. Reliable estimates of emissions associated with health care–related travel are essential for informing policy changes.

**Objective:**

To generate a comprehensive national estimate of carbon emissions due to patient health care–related travel in the US.

**Design, Setting, and Participants:**

This cross-sectional study used data from the 2022 National Household Travel Survey (NHTS), conducted from January 2022 to January 2023. Participants were selected using an address-based sample from the US Postal Service Delivery Sequence File. Participating households reported all trips taken within 24 hours by all household members aged 5 years or older. Approximate emissions per mile were obtained from typical vehicle emissions data provided by US government institutions. Data were analyzed between March 11 and May 29, 2024.

**Main Outcomes and Measures:**

Estimated annual CO_2_ equivalent (CO_2_e) emissions from patient health care–related travel per year, per patient, per trip, and per mile. A survey-weighted λ regression analysis was used to identify factors associated with higher CO_2_e emissions per trip. An alternative scenario analysis estimated reductions if 30% or 50% of private vehicle users switched to electric vehicles.

**Results:**

The sample included 16 997 participants with a weighted total of 3 506 325 536 US health care trips. Of these trips, 52.0% were reported by female travelers, 80.1% were made in urban areas, and 19.9% were made in rural areas. These trips accounted for 84 057 963 340 miles, resulting in weighted annual estimated emissions of 35.7 megatons (Mt) (95% CI, 27.5-43.9 Mt) CO_2_e. Each mile traveled generated an estimated 424 g (95% CI, 418-428 g) CO_2_e. Emissions per trip were higher (exponentiated coefficient [exp(β)], 2.19; 95% CI, 1.51-2.86; *P* < .001) for rural patients compared with urban patients. However, 69.3% of emissions were attributable to urban patients and 30.7% to rural patients. Patients with annual median household incomes of $50 000 to $99 999 generated higher trip emissions (exp[β], 1.92; 95% CI, 1.09-2.76; *P* = .003) compared with those with incomes of $25 000 or less. A 30% shift to electric vehicles was estimated to reduce health care–related carbon emissions to 27.6 Mt (95% CI, 20.7-34.6 Mt) CO_2_e, and a 50% shift was estimated to lower emissions to 22.3 Mt (95% CI, 16.0-28.6 Mt) CO_2_e.

**Conclusions and Relevance:**

This cross-sectional study estimated that annual patient health care–related travel in the US generated 35.7 Mt CO_2_e, which accounts for a small but important proportion of total health care–related emissions in the US. These findings are essential for informing health care policy decisions and suggest that strategies such as telehealth and the adoption of electric vehicles may contribute to a small but significant reduction in health care–related GHG emissions.

## Key Points

**Question:**

How much does health care–related patient travel contribute to CO_2_ equivalent (CO_2_e) emissions in the US?

**Findings:**

This cross-sectional study of data from 16 997 participants estimated that patient health care–related travel in the US generates approximately 35.7 megatons of CO_2_e annually.

**Meaning:**

This estimate of CO_2_e emissions from health care–related patient travel may be used for informing health care policy to reduce health care–related emissions.

## Teaser

This cross-sectional study uses data from the National Household Travel Survey to estimate annual greenhouse gas emissions associated with health care–related travel in the US.

## Introduction

The World Health Organization has declared that climate change is the greatest health threat to humanity.^1^ The effects of climate change on the environment are worsening and have a significant impact on various health outcomes.^2^ Climate change has been associated with cardiovascular, urogenital, and respiratory diseases as well as increased risks of lung, skin, gastrointestinal, and urogenital cancers.^3,4,5,6,7^ The impact of climate change on human health is significant, and the health care sector plays a critical role in managing these effects. However, the global health care sector itself is responsible for approximately 4.6% of worldwide greenhouse gas (GHG) emissions, highlighting an urgent need for strategies to reduce emissions within this sector.^8^

National-level assessments of emissions from the health care sector are essential for guiding health policy decisions. Growing efforts are being made to measure national health care emissions in the US and internationally.^9,10,11,12^ A study on health care–related emissions indicated that the US health care sector contributes 8.5% of the nation’s GHG emissions.^9^ Notably, private patient-related travel was not included in the analysis. An assessment of the carbon emissions from the National Health Service in England showed that the transportation of patients, visitors, and staff accounts for approximately 10% of the total emissions of the health service.^13^ Given the significant carbon footprint associated with transportation, there is increasing interest in quantifying emissions from health care–related travel. However, national estimates of carbon emissions from private patient-related travel in the US are lacking.

In this study, we sought to generate a comprehensive assessment of national carbon emissions due to patients’ health care–related travel, taking into account patient demographics, distance traveled, whether transportation was public or private, type of vehicle, and fuel type. We estimated both overall CO_2_ equivalent (CO_2_e) emissions due to health care travel and estimates on a per-patient, per-trip, and per-mile basis.

## Methods

### Data Sources and Study Design

This cross-sectional study used data from the National Household Travel Survey (NHTS) 2022, a nationally representative travel survey that captures daily passenger travel. The survey was conducted between January 2022 and January 2023, with participant travel days assigned for 7 days of the week over the 12-month period. Households were selected using an address-based sample derived from the US Postal Service Delivery Sequence File. Selected households were invited to participate in the survey through a mailed invitation letter. Participants could complete the survey online or by mail in English or Spanish. Each participating household reported all trips taken within 24 hours by all household members aged 5 years or older. Data collected included mode of transportation, vehicle type, vehicle fuel, and participant demographic and socioeconomic characteristics. Survey participants were required to select a single reason for their trip, with “health care trip” as an option covering both medical and dental services. Multiple trip purposes were not allowed. The overall response rate was 10.8%. To generate annual national representative estimates, the NHTS includes weights, reflecting selection probabilities for each household and adjusting for eligibility, nonresponse, undercoverage, geographic strata, day and month of response, and sociodemographic factors. The full list of variables (2022 NHTS Codebook, version 2.0^14^) and further information on the survey methods (2022 NHTS Users’ Guide, version 2.0^15^) are available online. The current study was conducted in accordance with the Strengthening the Reporting of Observational Studies in Epidemiology (STROBE) reporting guideline for cross-sectional studies.^16^ The survey data used in this study are deidentified and openly available online^17^; therefore, the study was deemed exempt by the Brigham and Women’s Hospital institutional review board.

To convert miles traveled for health care trips to CO_2_e, we calculated conversion factors for each type of transportation, type of vehicle, and type of fuel captured in the survey. We used US governmental sources to gather estimates of typical vehicle fuel economy, typical CO_2_e emissions per gallon of fuel, typical CO_2_e emissions for public transportation, and typical CO_2_e emissions for electric vehicles in the US. These estimates have been published by the US Department of Energy,^18^ the US Environmental Protection Agency,^19,20^ and the US Department of Transportation.^21^ A detailed description of the derivation of the conversion factors, including equations and specific sources, is provided in eTable 1 in Supplement 1. Conversion factors by transportation mode, vehicle type, and fuel type are shown in eTable 2 in Supplement 1. Data analysis was conducted between March 11 and May 29, 2024.

### Study Population

We limited our cohort to health care trips. No additional information on the type of visit or practitioner seen was available. If 2 members of the same household reported traveling together in a single vehicle to a health care facility, the trip was counted only once to avoid overestimating the mileage of shared journeys. Specifically, the trip reported by the passenger was excluded from the analysis. In addition, private trips for which the fuel type was not reported were excluded from the analysis.

### Measures

The main outcome measure was estimated annual CO_2_e emissions. Emissions were calculated for each health care trip by multiplying the miles traveled for each trip by individual conversion factors for each specific vehicle type, fuel type, and mode of transportation (Figure and eTables 1 and 2 in Supplement 1). Trip-level, person-level, and community-level covariates were categorized as described in Table 1. Categories of age, race and ethnicity, educational level, and annual median household income (MHI) were based on prior literature.^23,24^ Race and ethnicity, as self-reported on the NHTS, were included in the analysis because previous research has shown that there are racial and ethnic differences in the mode of transportation used to access health care.^23^ Categories were Hispanic, non-Hispanic Black, non-Hispanic White, and other (included American Indian or Alaska Native, Asian, and Native Hawaiian or Other Pacific Islander).

**Figure.**
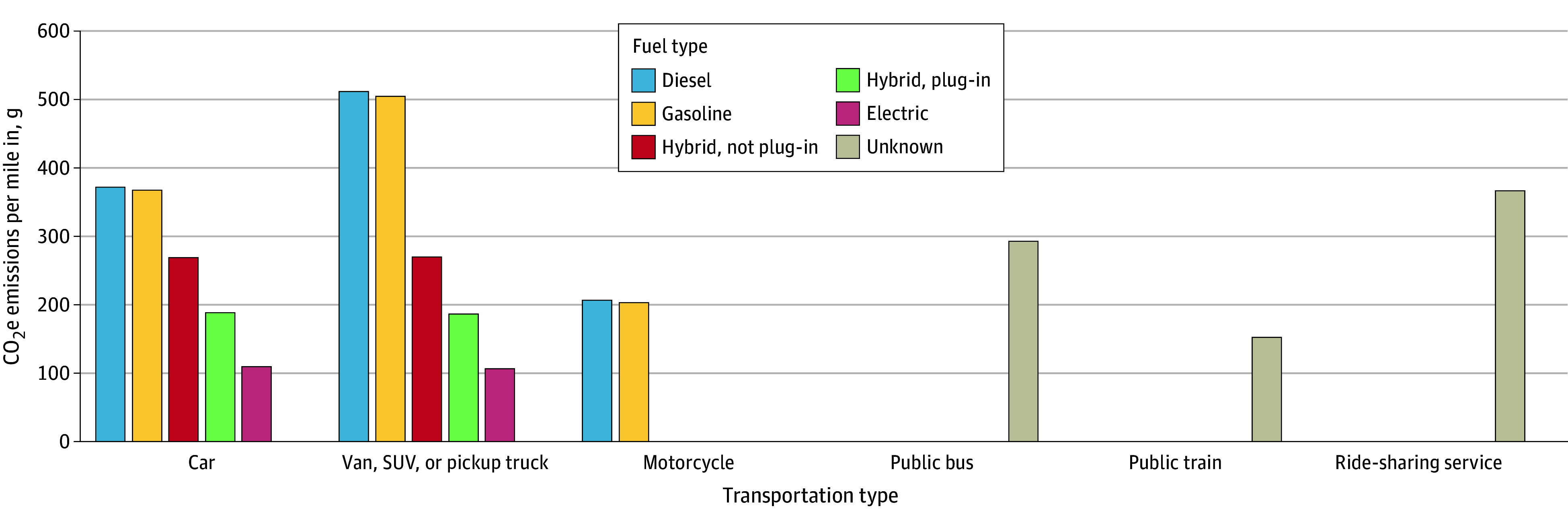
CO_2_ Equivalent (CO_2_e) Emissions by Type of Transportation, Type of Vehicle, and Type of Fuel for Patient Health Care Visits in the US SUV indicates sport utility vehicle.

**Table 1.** Baseline Characteristics of Patients’ Health Care Trips in the US in 2022

Sociodemographic characteristic	Health care trips, weighted No. (%)	Estimated CO_2_e emissions per health care trip, mean (95% CI), g	Distance traveled for health care trips, mean (95% CI), mi
Total	3 506 325 536 (100)	10 172.35 (8182.09-12 162.61)	23.97 (19.72-28.23)
Urbanicity^a^			
Urban	2 807 362 537 (80.1)	8 800.30 (6554.81-11 045.78)	21.16 (16.26-26.06)
Rural	698 962 999 (19.9)	15 683.18 (11 584.17-19 782.19)	35.27 (27.06-43.47)
Age, y			
≤25	159 910 661 (4.6)	17 532.18 (6167.59-28 896.76)	36.86 (12.83-60.88)
26-50	1 265 364 116 (36.1)	9722.72 (6495.93-12 949.50)	23.94 (16.15-31.73)
51-75	1 783 326 522 (50.9)	10 401.04 (7562.10-13 239.98)	24.01 (18.23-29.79)
≥76	297 724 237 (8.5)	6760.54 (3928.90-9592.17)	16.96 (9.18-24.75)
Sex			
Female	1 822 105 942 (52.0)	11 398.48 (8014.92-14 782.05)	26.71 (19.50-33.92)
Male	1 684 219 593 (48.0)	8845.84 (6963.87-10 727.80)	21.01 (16.88-25.14)
Race and ethnicity^b^			
Hispanic	644 038 536 (18.4)	12 541.62 (6940.86-18 142.38)	31.28 (18.92-43.65)
Non-Hispanic Black	332 772 557 (9.5)	6482.04 (4220.74-8743.35)	15.77 (9.61-21.93)
Non-Hispanic White	2 265 703 125 (64.6)	10 786.17 (8370.17-13 202.17)	24.69 (19.56-29.82)
Other^c^	263 811 317 (7.5)	3771.63 (2133.35-5409.91)	10.32 (6.65-13.99)
Educational level			
High school	1 453 235 420 (41.5)	12 230.75 (8007.80-16 453.71)	27.70 (18.84-36.56)
College or bachelor’s degree	1 391 608 163 (39.7)	9132.09 (7281.39-10 982.78)	23.13 (18.35-27.91)
Graduate degree	661 481 952 (18.9)	7838.66 (4190.71-11 486.61)	17.56 (10.17-24.96)
Annual MHI, USD			
≤25 000	411 886 839 (12.0)	5931.44 (2581.74-9281.15)	14.84 (8.18-21.50)
25 000-49 999	633 708 822 (18.4)	8976.51 (5318.56-12 634.45)	21.27 (13.00-29.53)
50 000-99 999	1 180 731 699 (34.3)	11 251.89 (7810.89-14 692.89)	27.20 (19.05-35.35)
≥100 000	1 220 764 006 (35.4)	10 082.86 (6945.44-13 220.28)	23.17 (17.02-29.31)
Census division			
New England	167 393 708 (4.8)	16 581.54 (458.79-32 704.29)	37.56 (4.33-70.80)
Middle Atlantic	553 116 754 (15.8)	7398.55 (3934.41-10 862.70)	19.05 (9.46-28.64)
East North Central	279 185 426 (8.0)	9161.99 (5087.93-13 236.04)	19.91 (11.64-28.17)
West North Central	280 711 735 (8.0)	12 303.36 (4391.62-20 215.09)	27.52 (9.43-45.61)
South Atlantic	618 392 738 (17.6)	8882.37 (6120.53-11 644.22)	21.85 (16.00-27.70)
East South Central	245 629 197 (7.0)	14 200.02 (3128.96-25 271.08)	30.73 (7.52-53.94)
West South Central	543 252 350 (15.5)	9192.13 (5268.50-13 115.76)	21.45 (11.27-31.63)
Mountain	317 939 597 (9.1)	13 329.13 (5519.42-21 138.83)	30.27 (13.33-47.20)
Pacific	500 704 031 (14.3)	9138.82 (3376.82-14 900.83)	23.20 (10.83-35.57)

Abbreviations: CO_2_e, CO_2_ equivalent; MHI, median household income.

^a^ Urban or rural classification of participant households was determined using the US Census Bureau 2020 TIGER/Line Shapefile classification.^22^

^b^ Self-reported by participants of the National Household Travel Survey.

^c^ Includes American Indian or Alaska Native, Asian, and Native Hawaiian or Other Pacific Islander.

### Statistical Analysis

The unit of analysis was an individual patient’s health care trip. Each household that participated in the survey was assigned a unique 10-digit household identification number. To weight the sample, we used the household identification number as the primary sampling unit and applied the 7-day trip weights to estimate nationwide health care trips throughout 2022. Previous literature assumed that CO_2_ emissions follow a λ distribution.^25^ To assess the association of patient- and community-level factors with CO_2_e emissions per health care trip, we conducted a λ regression analysis. We adjusted for urban or rural residence (using the US Census Bureau 2020 TIGER/Line Shapefile classification^22^), US region, age, sex, race and ethnicity, educational level, and MHI to identify factors associated with CO_2_e emissions per health care trip. We conducted a sensitivity analysis using an assumed emission of 1g CO_2_e per mile traveled by bicycle or foot, which yielded consistent results. To enhance the interpretability of the model results, we exponentiated the coefficients. The exponentiated coefficients (exp[β]) indicate the ratio of means for a 1-unit increase in a covariate. Subsequently, we performed a postestimation analysis using marginal analysis to calculate the mean CO_2_e emissions per health care trip for different patient subgroups. Finally, we conducted an alternative scenario analysis to estimate the carbon emissions if 30% and 50% of patients using private vehicles for health care visits chose to drive electric vehicles. To model these scenarios, we randomly assigned values between 0 and 1 to patients using private vehicles for their health care trips. Based on these assignments, we selected 30% and 50% of private trips to be made with electric vehicles and recalculated total emissions accordingly.

Statistical analysis was performed using Stata/BE, version 17.0 (StataCorp LLC). Significance tests were 2-sided, and *P* < .05 was considered statistically significant.

## Results

### Trip, Patient, and Community Baseline Characteristics

The NHTS 2022 collected data from 7893 households and 16 997 participants who reported a total of 31 074 trips. This sample represents weighted estimates of 127 544 707 households and 305 560 925 participants who collectively made 231 715 789 606 trips nationwide in 2022. Of the total trips recorded, 2.2% were trips to health care facilities. After excluding 155 passenger trips and 2 trips with missing information on fuel type, our study cohort included 526 trips to health care facilities, representing a weighted total of 3 506 325 536 annual trips to health care facilities in the US in 2022. A total of 52.0% of weighted health care trips were reported by female travelers and 48.0% by male travelers. In all, 9.5% of weighted health care trips were reported by Black participants, 18.4% by Hispanic participants, 64.6% by White participants, and 7.5% by participants of other race and ethnicity. The majority of weighted health care trips (80.1%) were taken by patients living in urban areas compared with rural areas (19.9%) (Table 1).

### Carbon Emission of Transportation to Health Care Facilities in the US in 2022

The weighted sum of miles traveled annually for health care visits in the US in 2022 was 84 057 963 340 miles, resulting in a weighted total annual CO_2_e emissions estimate for health care trips of 35.7 megatons (Mt) (95% CI, 27.5-43.9 Mt). The mean distance traveled for health care trips per person per year was 275.1 (95% CI, 215.1-335.1) miles per person per year, resulting in mean estimated emissions of 116.7 kg (95% CI, 89.9-143.6 kg) CO_2_e for health care trips per person per year in the US in 2022. The mean estimated amount of CO_2_e emitted per trip was 10 172 g (95% CI, 8182-12 163 g). The weighted mean estimates of carbon emissions per mile for health care visits in 2022 were 424 g (95% CI, 418-428 g) CO_2_e nationwide, 416 g (95% CI, 406-421 g) CO_2_e in urban areas, and 445 g (95% CI, 430-453 g) CO_2_e in rural areas (eTable 3 in Supplement 1).

### Mode of Transportation and Type of Fuel

In this study, 43.5% of health care trips were taken by car, 33.0% by sport utility vehicle or crossover, and 8.0% by pickup truck. Public transportation accounted for only 6.0% of health care trips and was used primarily by urban residents. Specifically, 99.5% of public bus trips to health care facilities were made by individuals living in urban areas compared with 0.5% by those living in rural areas (Table 2). Among health care–related trips made by private vehicles, 93.3% were powered by gasoline, 0.6% by diesel, 0.3% by plug-in hybrid vehicles, 1.7% by electric vehicles, and 4.1% by non–plug-in hybrid vehicles. The distribution of private health care trips by vehicle fuel type is provided in eTable 4 in Supplement 1.

**Table 2.** Weighted Number of Patients’ Health Care Trips by Mode of Transportation and Sociodemographic Characteristics

Characteristic	Health care trips, No. (%) (N = 3 506 325 536)										
	Car	Van	SUV or crossover	Pickup truck	Motorcycle	Bus	Train	Ride-sharing	Bicycle	Walked
Total	1 524 057 947 (43.5)	193 678 833 (5.5)	1 157 558 604 (33.0)	279 804 375 (8.0)	1 604 237 (<0.1)	205 047 781 (5.8)	6 774 141 (0.2)	63 436 359 (1.8)	33 998 293 (1.0)	40 364 965 (1.2)
Urbanicity^a^										
Urban	1 189 361 794 (78.0)	149 822 470 (77.4)	956 556 011 (82.6)	165 075 736 (59.0)	1 604 237 (100)	204 082 965 (99.5)	6 774 141 (100)	59 721 916 (94.1)	33 998 293 (100)	40 364 965 (100)
Rural	334 696 153 (22.0)	43 856 354 (22.6)	201 002 594 (17.4)	114 728 639 (41.0)	0	964 816 (0.5)	0	3 714 443 (5.9)	0	0
Age, y										
≤25	38 692 657 (2.5)	0	78 573 186 (6.8)	13 211 319 (4.7)	0	0	595 786 (67.8)	714 443 (5.9)	1 123 270 (62.1)	0
26-50	481 528 649 (31.6)	38 925 217 (20.1)	519 739 152 (44.9)	30 131 264 (10.8)	0	166 765 060 (81.3)	2 178 356 (32.2)	2 379 111 (3.8)	0	23 717 308 (58.8)
51-75	839 368 282 (55.1)	150 653 181 (77.8)	492 828 186 (42.6)	195 159 022 (69.7)	1 604 237 (100)	30 646 542 (14.9)	0	56 419 417 (89.0)	0	16 647 658 (41.2)
≥76	164 468 360 (10.8)	4 100 436 (2.1)	66 418 080 (5.7)	41 302 770 (14.8)	0	7 636 180 (3.7)	0	923 389 (1.5)	12 875 023 (37.9)	0
Sex										
Female	800 418 225 (52.5)	124 419 659 (64.2)	656 455 566 (56.7)	58 368 947 (20.9)	0	134 064 702 (65.4)	2 178 356 (32.2)	25 216 542 (39.8)	12 875 023 (37.9)	8 108 923 (20.1)
Male	723 639 722 (47.5)	69 259 174 (35.8)	501 103 038 (43.3)	221 435 428 (79.1)	1 604 237 (100)	70 983 079 (34.6)	4 595 786 (67.8)	38 219 818 (60.2)	21 123 270 (62.1)	32 256 043 (79.9)
Race and ethnicity^b^										
Hispanic	370 991 486 (24.3)	59 723 892 (30.8)	161 109 450 (13.9)	11 465 192 (4.1)	0	3 711 471 (1.8)	4 595 786 (67.8)	32 441 260 (51.1)	0	0
Non-Hispanic Black	138 213 410 (9.1)	3 088 496 (1.6)	153 743 321 (13.3)	7 206 654 (2.6)	0	13 968 612 (6.8)	0	16 552 056 (26.1)	0	0
Non-Hispanic White	973 416 191 (63.9)	130 866 445 (67.6)	806 327 409 (69.7)	250 481 007 (89.5)	1 604 237 (100)	34 571 251 (16.9)	0	5 592 905 (8.8)	22 478 716 (66.1)	40 364 965 (100)
Other^c^	41 436 852 (2.7)	0	36 378 424 (3.1)	10 651 523 (3.81)	0	152 796 448 (74.5)	2 178 356 (32.2)	8 850 138 (14.0)	11 519 577 (33.9)	0
Educational level										
High school	558 763 209 (36.7)	109 529 239 (56.6)	382 116 463 (33.0)	161 597 869 (57.8)	0	170 012 413 (82.9)	0	46 052 083 (72.6)	21 123 270 (62.1)	4 040 876 (1.0)
College or bachelor’s degree	734 554 546 (48.2)	51 826 872 (26.8)	453 738 509 (39.2)	96 664 264 (34.5)	1 604 237 (100)	25 570 673 (12.5)	4 595 786 (67.8)	11 325 193 (17.9)	0	11 728 084 (29.1)
Graduate degree	230 740 192 (15.1)	32 322 722 (16.7)	321 703 633 (27.8)	21 542 242 (7.7)	0	9 464 695 (4.6)	2 178 356 (32.2)	6 059 084 (9.6)	12 875 023 (37.9)	24 596 006 (60.9)
Annual MHI, USD										
≤25 000	159 536 089 (10.7)	36 233 110 (18.7)	38 829 946 (3.4)	15 573 971 (5.6)	0	130 096 660 (63.4)	0	22 927 206 (36.1)	0	8 689 856 (21.5)
25 000-49 999	276 354 748 (18.5)	55 323 069 (28.6)	164 674 863 (14.6)	62 825 194 (22.5)	0	66 935 499 (32.6)	0	3 714 443 (5.9)	0	3 881 005 (9.6)
50 000-99 999	579 716 316 (38.8)	50 190 956 (25.9)	388 496 409 (34.5)	98 792 675 (35.3)	0	8 015 622 (3.9)	4 595 786 (67.8)	28 445 221 (44.8)	22 478 716 (66.1)	0
≥100 000	479 228 148 (32.1)	51 931 698 (26.8)	535 545 862 (47.5)	102 612 535 (36.7)	1 604 237 (100)	0	2 178 356 (32.2)	8 349 490 (13.2)	11 519 577 (33.9)	27 794 104 (68.9)
Census division										
New England	93 121 764 (6.1)	1 134 566 (0.6)	41 935 865 (3.6)	18 899 101 (6.8)	0	0	4 595 786 (67.8)	7 706 627 (12.1)	0	0
Middle Atlantic	250 677 991 (16.4)	8 803 821 (4.6)	113 840 074 (9.8)	38 331 283 (13.7)	1 604 237 (100)	78 008 983 (38.0)	2 178 356 (32.2)	23 495 178 (37.0)	11 519 577 (33.9)	24 657 255 (61.1)
East North Central	95 122 343 (6.2)	30 060 099 (15.5)	110 561 816 (9.6)	33 773 169 (12.1)	0	7 288 889 (3.6)	0	2 379 111 (3.8)	0	0
West North Central	98 671 694 (6.5)	2 879 565 (1.5)	148 107 995 (12.8)	18 177 458 (6.5)	0	0	0	0	12 875 023 (37.9)	0
South Atlantic	353 434 385 (23.2)	30 874 143 (15.9)	180 581 082 (15.6)	37 914 832 (13.6)	0	6 521 923 (3.2)	0	7 298 539 (11.5)	0	1 767 834 (4.4)
East South Central	88 395 113 (5.8)	26 319 215 (13.6)	99 526 230 (8.6)	30 423 823 (10.9)	0	964 816 (0.5)	0	0	0	0
West South Central	193 805 426 (12.7)	64 100 771 (33.1)	215 958 925 (18.7)	44 341 585 (15.8)	0	3 424 011 (1.7)	0	20 266 499 (31.9)	0	1 355 133 (3.4)
Mountain	158 839 087 (10.4)	21 477 859 (11.1)	114 485 475 (9.9)	23 137 176 (8.3)	0	0	0	0	0	0
Pacific	191 990 144 (12.6)	8 028 794 (4.2)	132 561 143 (11.5)	34 805 948 (12.4)	0	108 839 160 (53.1)	0	2 290 406 (3.6)	9 603 693 (28.2)	12 584 744 (31.2)

Abbreviations: MHI, median household income; SUV, sport utility vehicle.

^a^ Urban or rural classification of participant households was determined using the US Census Bureau 2020 TIGER/Line Shapefile classification.^22^

^b^ Self-reported by participants of the National Household Travel Survey.

^c^ Includes American Indian or Alaska Native, Asian, and Native Hawaiian or Other Pacific Islander.

### Annual CO_2_e Emissions for Patients’ Health Care Trips by Sociodemographic Factors

Annual CO_2_e emissions from patients’ health care–related travel in the US were estimated at 35.7 Mt (95% CI, 27.5-43.9 Mt). Of these emissions, 69.3% were attributed to patients living in urban areas, while 30.7% were attributed to patients living in rural areas. Patients aged 51 to 75 years contributed an estimated 18.5 Mt (95% CI, 12.6-24.5 Mt) CO_2_e, or 52.0% of the total emissions across all age groups. Emissions estimates for health care–related travel by MHI groups were as follows: patients with an MHI of $25 000 or less generated 2.4 Mt (95% CI, 0.8-4.1 Mt) CO_2_e (7.2% of the total), patients with an MHI of $25 000 to $49 999 generated 5.7 Mt (95% CI, 3.1-8.3 Mt) CO_2_e (16.9%), patients with an MHI of $50 000 to $99 999 generated 13.3 Mt (95% CI, 7.8-18.8 Mt) CO_2_e (39.4%), and patients with an MHI of $100 000 or more generated 12.3 Mt (95% CI, 7.2-17.4 Mt) CO_2_e (36.5%) (Table 3).

**Table 3.** Weighted Annual CO_2_e Emissions for Patients’ Health Care Trips by Sociodemographic Characteristics

Characteristic	Annual estimated CO_2_e emissions, megatons (95% CI) [% of total]
Urbanicity^a^	
Urban	24.7 (17.2 to 32.2) [69.3]
Rural	11.0 (7.1 to 14.9) [30.7]
Age, y	
≤25	2.8 (−0.7 to 6.3) [7.9]
26-50	12.3 (7.5 to 17.1) [34.5]
51-75	18.5 (12.6 to 24.5) [52.0]
≥76	2.0 (1.0 to 3.1) [5.6]
Sex	
Female	20.8 (13.2 to 28.3) [58.2]
Male	14.9 (11.0 to 18.8) [41.8]
Educational level	
High school	17.8 (10.5 to 25.0) [49.8]
College or bachelor’s degree	12.7 (9.4 to 16.0) [35.6]
Graduate degree	5.2 (2.3 to 8.1) [14.5]
Annual MHI, USD	
≤25 000	2.4 (0.8 to 4.1) [7.2]
25 000-49 999	5.7 (3.1 to 8.3) [16.9]
50 000-99 999	13.3 (7.8 to 18.8) [39.4]
≥100 000	12.3 (7.2 to 17.4) [36.5]
Census division	
New England	2.8 (−0.6 to 6.2) [7.8]
Middle Atlantic	4.1 (1.2 to 7.0) [11.5]
East North Central	2.6 (1.1 to 4.1) [7.2]
West North Central	3.5 (1.1 to 5.8) [9.7]
South Atlantic	5.5 (3.3 to 7.7) [15.4]
East South Central	3.5 (0.2 to 6.8) [9.8]
West South Central	5.0 (1.9 to 8.1) [14.0]
Mountain	4.2 (0.5 to 8.0) [11.9]
Pacific	4.6 (1.4 to 7.8) [12.8]

Abbreviations: CO_2_e, CO_2_ equivalent; MHI, median household income.

^a^ Urban or rural classification of participant households was determined using the US Census Bureau 2020 TIGER/Line Shapefile classification.^22^

### Patient Characteristics Associated With Higher CO_2_e Emissions

Patients living in a rural area were estimated to emit more CO_2_e per health care trip than patients living in an urban area (exp[β], 2.19; 95% CI, 1.51-2.86; *P* < .001). Patients with different income levels had different estimated CO_2_e emissions per health care trip, with greater emissions seen among patients with higher incomes. Those with an MHI between $50 000 and $99 999 had higher emissions (exp[β], 1.92; 95% CI, 1.09-2.76; *P* = .003) compared with patients whose MHI was $25 000 or less (Table 4).

**Table 4.** Results of λ Regression Analysis of Carbon Emissions Per Patient Health Care Trip in the US

Characteristic	Exp[β] (95% CI)^a^	*P* value
Urbanicity^b^		
Urban	1 [Reference]	NA
Rural	2.19 (1.51 to 2.86)	<.001
Age, y		
≤25	1 [Reference]	NA
26-50	0.93 (0.26 to 1.60)	.84
51-75	0.80 (0.27 to 1.32)	.50
≥76	0.63 (0.13 to 1.12)	.25
Sex		
Female	1.06 (0.76 to 1.37)	.68
Male	1 [Reference]	NA
Race and ethnicity^c^		
Hispanic	1.46 (0.81 to 2.12)	.10
Non-Hispanic Black	0.75 (0.42 to 1.08)	.20
Non-Hispanic White	1 [Reference]	NA
Other^d^	0.47 (0.24 to 0.71)	.003
Educational level		
High school	1 [Reference]	NA
College or bachelor’s degree	0.86 (0.59 to 1.13)	.34
Graduate degree	0.76 (0.47 to 1.04)	.15
Annual MHI, USD		
≤25 000	1 [Reference]	NA
25 000-49 999	1.67 (0.85 to 2.48)	.04
50 000-999 999	1.92 (1.09 to 2.76)	.003
≥100 000	1.72 (0.93 to 2.50)	.02
Census division		
New England	1 [Reference]	NA
Middle Atlantic	0.37 (0.02 to 0.73)	.04
East North Central	0.57 (0.04 to 1.09)	.23
West North Central	0.62 (−0.03 to 1.27)	.38
South Atlantic	0.49 (0.06 to 0.92)	.11
East South Central	0.39 (0.00 to 0.77)	.06
West South Central	0.47 (0.04 to 0.90)	.11
Mountain	0.64 (−0.00 to 1.28)	.38
Pacific	0.67 (0.04 to 1.30)	.40

Abbreviations: exp[β], exponentiated coefficient; MHI, median household income; NA, not applicable.

^a^ The exp[β] indicates the ratio of means for a 1-unit increase in a covariate.

^b^ Urban or rural classification of participant households was determined using the US Census Bureau 2020 TIGER/Line Shapefile classification.^22^

^c^ Self-reported by participants of the National Household Travel Survey.

^d^ Includes American Indian or Alaska Native, Asian, and Native Hawaiian or Other Pacific Islander.

### Mean Amount of CO_2_e Emitted for a Health Care Trip by Different Subgroups

A health care trip made by an urban resident was estimated to emit 7982 g (95% CI, 6558-9406 g) CO_2_e, while a health care trip made by a rural resident was estimated to emit 17 454 g (95% CI, 12 349-22 558 g) CO_2_e. A health care trip made by a patient with an MHI of $25 000 or less was estimated to emit 5723 g (95% CI, 3513-7933 g) CO_2_e, while a health care trip made by a patient with an MHI of $100 000 or more was estimated to emit 9833 g (95% CI, 7555-12 111 g) CO_2_e (eFigure in Supplement 1).

### Alternative Scenario Analysis

In the alternative scenario analysis, shifting 30% of private vehicle use to electric vehicles reduced estimated health care–related carbon emissions from 35.7 Mt (95% CI, 27.5-43.9 Mt) CO_2_e to 27.6 Mt (95% CI, 20.7-34.6 Mt) CO_2_e. A 50% shift further decreased estimated emissions to 22.3 Mt (95% CI, 16.0-28.6 Mt) CO_2_e.

## Discussion

We used a nationally representative travel survey of US households to examine the total carbon emissions from patients’ health care trips in the US in 2022 and estimated CO_2_e emissions on a per-patient, per-trip, and per-mile basis. We found that the typical US individual traveled 275 miles annually for health care–related visits, resulting in estimated emissions of 424 g CO_2_e per mile traveled. Patients’ health care trips in the US were responsible for total annual estimated emissions of 35.7 Mt CO_2_e in 2022, which would account for 6.4% of the 554 Mt CO_2_e recently estimated as the total annual GHG emissions from the health care sector in the US.^9^ To put this into context, these health care trip emissions are equivalent to GHG emissions from the electricity use of 7 million homes for 1 year or the emissions from 9 coal-fired power plants over the same period.^26^

Assessing the environmental impacts of the health care sector is crucial for developing effective strategies to mitigate its emissions. A growing body of literature has estimated health care–related emissions at both national and global levels, primarily using monetary expenditure analyses.^9,10,11,12^ However, emissions from private patient-related health care travel are often excluded from these assessments because the associated costs are incurred privately and are not included in national health care expenditures. Notably, a UK study estimated the national emissions from the National Health Service in England, including emissions from staff, patient, and visitor travel.^13^ To account for patient and visitor travel emissions, the authors incorporated data from the National UK Travel Survey.^27^ They estimated that in 2019, annual travel-related emissions from patients, visitors, and staff commuting amounted to 2.5 Mt CO_2_e, representing 10% of total health care emissions. In the present study, we estimated that patient travel–related emissions in the US generated 35.7 Mt CO_2_e in 2022. Our estimates of patient travel–related health care emissions provide a valuable foundation for future research on national health care emissions in the US.

Our analysis indicated that 1.7% of national health care–related trips made by private vehicles were conducted using electric vehicles. The adoption of electric vehicles has increased rapidly over the past decade. Future projections consistently predict continued growth in electric vehicle uptake, but estimates vary widely across different sources.^28^ In 2023, the US government set ambitious goals to accelerate the adoption of electric vehicles, aiming for 50% of all new vehicle sales to be electric by 2030.^29^ In our alternative scenario analysis, we estimated the potential reduction in carbon emissions if a specific percentage of private health care trips was conducted using electric vehicles. We estimated that annual national emissions from patient health care–related travel could decrease from 35.7 Mt CO_2_e to 27.6 Mt CO_2_e if 30% of patients using private vehicles for health care–related travel used electric vehicles. Emissions could further decline to 22.3 Mt CO_2_e if 50% used electric vehicles. However, the carbon emissions from electric vehicles are highly dependent on the electric mix consumed during their production and use phase.^30^ Predictions of US electricity grid projections were not included in our alternative scenario analysis; therefore, the estimates should be interpreted with considerable caution.

Another potential strategy for reducing emissions from health care–related travel that has gained attention in recent years is telehealth. Most studies assessing the impacts of telehealth on care delivery have focused on the convenience of telehealth and the potential improvements in access to care, quality of care, and equity.^31,32^ Some studies have indicated that providing telehealth consultations instead of in-person visits significantly reduces health care–related emissions.^33,34^ Most commonly, carbon assessments related to telehealth are done by calculating the travel distance saved by telehealth visits and multiplying it by a conversion factor. However, systematic reviews investigating the reduction of emissions through telehealth report significant heterogeneity in the methods used to estimate emissions from travel to health care facilities.^35,36^ In our analysis, we estimated the national mean CO_2_e emission for traveling 1 mile for a health care visit to be 424 g CO_2_e. Additionally, we estimated the mean emissions for driving 1 mile for a health care visit in urban areas at 416 g CO_2_e and in rural areas at 445 g CO_2_e. These estimates provide a valuable foundation for future research exploring the potential national carbon emission savings achievable through telehealth in the US.

We investigated sociodemographic factors associated with increased CO_2_e emissions per health care trip and found that patients living in rural areas generated more than twice the carbon emissions per health care trip than those living in urban areas. However, 69.3% of the annual estimated CO_2_e emissions were attributable to patients in urban areas, while only 30.7% came from patients in rural areas. The higher total emissions observed among urban patients may be attributable to the significantly higher proportion of patients living in urban areas (80.1%) compared with rural areas (19.9%). Previous studies have highlighted numerous benefits of telehealth in rural areas, including increased access to care and reduced costs for both patients and practitioners.^32,37^ Given the higher carbon emissions of health care trips by patients in rural areas, our findings underscore another potential rationale for expanding telehealth access in these areas.

### Limitations

Our study has some limitations. We calculated conversion factors based on average fuel economy and average emissions estimates from several different US government sources. This approach could carry imprecision due to inherent variability. Therefore, the estimated carbon emissions should be considered approximate estimates rather than precise measurements. However, to minimize inherent variability in the underlying data, we meticulously searched governmental sources to ensure that the emissions data for each type of transportation and fuel type incorporated only the use phase. This approach excludes the production phase of the vehicle, providing comparability in our estimates. Furthermore, we have provided detailed descriptions of the conversion factors used to translate miles traveled for health care visits into carbon emissions, along with the underlying assumptions and the sources from which the data were derived to ensure the reproducibility of our results (eTable 1 in Supplement 1).

Some fuel economy and emissions estimates provided by government institutions have not been updated recently. However, the most recent available estimates were used to calculate fuel and vehicle conversion factors. Another limitation of this study is that the vehicle and fuel type assumptions were based on US estimates, which means that the conversion factors are only applicable to US health care trips. Additionally, this cross-sectional study used survey data relying on a sample of respondents who agreed to participate, and findings from this population might not be generalizable. However, the survey used a complex weighting system that adjusted for the selection probabilities of each household and accounted for factors such as eligibility, nonresponse, undercoverage, geographic stratification, the day and month of response, and sociodemographic characteristics.^15^ In addition, our study did not account for transportation of health care practitioners and clinical staff and did not include other environmental impacts of health care–related travel, such as air pollution, which also affects human health.

## Conclusions

In this cross-sectional study, we estimated that annual national health care–related patient travel in the US generated 35.7 Mt CO_2_e, which would account for 6.4% of the total national health care–related emissions estimated elsewhere.^9^ These findings provide estimates of emissions attributable to health care–related patient travel, which are essential for informing health care policy decisions. Greater adoption of electric vehicles for private transportation and changes in health care delivery, such as the increased use of telemedicine, are potential avenues to reduce GHG emissions from the health care sector.

## Notes

Supplement 1. **eTable 1.** Calculation of CO2e Emissions and Sources **eTable 2.** Conversion Factors to Convert Miles Driven With a Specific Mode of Transportation to CO2e **eTable 3.** Distance and Carbon Emission Estimates for Patients’ Health Care Trips in the US in 2022 **eTable 4.** Weighted Number of Patients’ Health Care Trips by Fuel Type for Private Vehicles Used for Health Care Trips and by Sociodemographic Characteristics **eFigure.** Carbon Emissions per Health Care Trip Across Different Patient Subgroups

Supplement 2.
Data Sharing Statement
