# Supplementary material for: Carbon Emissions From Patient Travel for Health Care
Source: JAMA Netw Open. 2025 Mar 31;8(3):e252513. doi: 10.1001/jamanetworkopen.2025.2513 (PMC11959441; doi:10.1001/jamanetworkopen.2025.2513)
Supplement: Supplement 1. — eTable 1. Calculation of CO2e Emissions and Sources eTable 2. Conversion Factors to Convert Miles Driven With a Specific Mode of Transportation to CO2e eTable 3. Distance and Carbon Emission Estimates for Patients’ Health Care Trips in the US in 2022 eTable 4. Weighted Number of Patients’ Health Care Trips by Fuel Type for Private Vehicles Used for Health Care Trips and by Sociodemographic Characteristics eFigure. Carbon Emissions per Health Care Trip Across Different Patient Subgroups [file jamanetwopen-e252513-s001.pdf]

Supplementary Online Content

Zurl H, Qian Z, Stelzl DR, et al. Carbon emissions from patient travel for health care. *JAMA Netw Open*. 2025;8(3):e252513. doi:10.1001/jamanetworkopen.2025.2513

eTable 1. Calculation of CO_2e Emissions and Sources

eTable 2. Conversion Factors to Convert Miles Driven With a Specific Mode of Transportation to CO_2e

eTable 3. Distance and Carbon Emission Estimates for Patients' Health Care Trips in the US in 2022

eTable 4. Weighted Number of Patients' Health Care Trips by Fuel Type for Private Vehicles Used for Health Care Trips and by Sociodemographic Characteristics

eFigure. Carbon Emissions per Health Care Trip Across Different Patient Subgroups

This supplementary material has been provided by the authors to give readers additional information about their work.

© 2025 Zurl H et al. *JAMA Network Open*.

**eTable 1: Calculation of CO_2e Emissions and Sources**	**Car**	**Van SUV/Crossover Pickup Truck**	**Motorcycle**	**Bus**	**Train**	**Ride-Sharing**
**Vehicle fuel economy (Gasoline)** (Source: US DOE, https://afdc.energy.gov/data/10310)	24.4mpg	17.8mpg	44.0mpg	-	-	24.4mpg
**CO_2 emitted per gallon (Gasoline)** (Source: US EPA, https://www.epa.gov/greenvehicles/greenhouse-gas-emissions-typical-passenger-vehicle#driving)	8 887g CO_2/gallon	8 887g CO_2/gallon	8 887g CO_2/gallon	-	-	8 887g CO_2/gallon
**CO_2 emissions per mile (Gasoline)**	364.2g CO_2/mile	499.3g CO_2/mile	202g CO_2/mile	-	-	364.2g CO_2/mile
**CO_2e emission per mile (Gasoline)** Ratio of emissions of greenhouse gases other than CO_2: 1.01 (Source: US EPA, https://www3.epa.gov/carbon-footprint-calculator/)	367.9g CO_2e/mile	504.3g CO_2e/mile	204g CO_2e/mile	-	-	367.9g CO_2e/mile
**Vehicle fuel economy (Diesel)** (Source: US Department of Energy, https://afdc.energy.gov/data/10310)	27.6mpg	20.1mpg	49.7mpg	-	-	-
**CO_2 emitted per gallon (Diesel)** (Source: US EPA, https://www.epa.gov/greenvehicles/greenhouse-gas-emissions-typical-passenger-vehicle#driving)	10 180g CO_2/gallon	10 180g CO_2/gallon	10 180g CO_2/gallon	-	-	-
**CO_2 emissions per mile (Diesel)**	368.8g CO_2/mile	506.5g CO_2/mile	204.8g CO_2/mile	-	-	-
**CO_2e emission per mile (Diesel)** Ratio of emissions of greenhouse gases other than CO_2: 1.01 (Source: US EPA, https://www3.epa.gov/carbon-footprint-calculator/)	372.5g CO_2e/mile	511.5g CO_2e/mile	206.9g CO_2e/mile	-	-	-
**CO_2e emissions per mile Electric Vehicle** 2727 pounds CO_2e per vehicle per year, 11579 average annual vehicle miles (Source: US DOE, https://afdc.energy.gov/vehicles/electric_emissions.html)	107g CO_2e/mile	107g CO_2e/mile	-	-	-	-

© 2025 Zurl H et al. *JAMA Network Open*.

eTable 1: Calculation of CO_2e Emissions and Sources (continued)						
**CO_2e emissions per mile Plug-in Hybrid Vehicle** 4763 pounds CO_2e per vehicle per year, 11579 average annual vehicle miles (Source: US DOE, https://afdc.energy.gov/vehicles/electric_emissions.html)	187g CO_2e/mile	187g CO_2e/mile	-	-	-	-
**CO_2e emissions per mile Non-Plug in Hybrid Vehicle** 6898 pounds CO_2e per vehicle per year, 11579 average annual vehicle miles (Source: US DOE, https://afdc.energy.gov/vehicles/electric_emissions.html)	270g CO_2e/mile	270g CO_2e/mile	-	-	-	-
**CO_2 emissions per passenger mile[a]** (Source: US Department of Transportation, https://www.transit.dot.gov/sites/fta.dot.gov/files/docs/PublicTransportationsRoleInRespondingToClimateChange2010.pdf)				0.64 pounds/ mile= 290.3g CO_2/mile	0.33 pounds/ mile = 149.9g CO_2/mile	-
**CO_2e emissions per passenger mile[*]** Ratio of emissions of greenhouse gases other than CO_2: 1.01 (Source: US EPA, https://www3.epa.gov/carbon-footprint-calculator/)				293g CO_2e/mile	151.4g CO_2e/mile	-

This table presents the conversion factors used to translate miles traveled for healthcare visits into carbon emissions, along with the estimates behind their calculations and the respective sources from which the data were derived. Abbreviations: US DOE: United States Department of Energy, US EPA: United States Environmental Protection Agency, CO_2: Carbon Dioxide, CO_2e: Carbon Dioxide Equivalent, mpg: miles per gallon, g: gram. ***Example for calculating the CO_2e emissions per mile driven with an average gasoline-powered car:*** *The average vehicle fuel economy for a gasoline-powered car is 24.4 mpg, and the average CO_2 emitted per gallon of gasoline is 8 887g. 8 887g of CO_2 is emitted for driving 24.4 miles. 1/24.4 x 8 887= 364.2 g of CO_2 is emitted for driving one mile. The ratio of emissions of greenhouse gases other than CO_2 is 1.01. 364.2g x 1.01 = 367.9g CO_2e. 367.9g CO_2e is emitted for driving 1 mile with an average gasoline-powered car.* ***Example for calculating the CO_2e emissions per mile driven with an electric vehicle:*** *The annual average vehicle emissions of an electric vehicle are 2727 pounds per vehicle per year. The average annual distance traveled is 11 579. 2 727 pounds CO_2e/11 579miles= 0.107 kg/mile= 107g/mile. [a]CO_2e emissions from public transportation are calculated on a per-passenger-mile basis, reflecting the shared nature of these services. In contrast, emissions from private transport are calculated per vehicle.*

© 2025 Zurl H et al. *JAMA Network Open*.

eTable 2: Conversion Factors to Convert Miles Driven With a Specific Mode of Transportation to CO_2e	
**Vehicle Type NHTS**	**Amount of CO_2e emitted per passenger mile**
**Car**	Gasoline: 368 g CO_2e/mile<br>Diesel: 373 g CO_2e/mile<br>Plug-in hybrid: 187 g CO_2e/mile<br>Electric only: 107 g CO_2e/mile<br>Hybrid, gas/electric, non plug-in: 270 g CO_2e/mile
**Van, SUV/Crossover, Pickup truck**	Gasoline: 504 g CO_2e/mile<br>Diesel: 512 g CO_2e/mile<br>Plug-in hybrid: 187 g CO_2e/mile<br>Electric only: 107 g CO_2e/mile<br>Hybrid, gas/electric, non plug-in: 270 g CO_2e/mile
**Motorcycle**	Gasoline: 204 g CO_2e/mile<br>Diesel: 207 g CO_2e/mile
**Bus**	293 g CO_2e/mile
**Train**	151 g CO_2e/mile
**Ride-sharing services**	Gasoline: 368 g CO_2e/mile<br>Diesel: 373 g CO_2e/mile
**Bicycle**	0 g CO_2e/mile
**Walked**	0 g CO_2e/mile

© 2025 Zurl H et al. *JAMA Network Open*.

eTable 3: Distance and Carbon Emission Estimates for Patients' Health Care Trips in the US in 2022	
Nationwide weighted sum of miles traveled for healthcare trips in the US in 2022	84 057 963 340 miles
Nationwide weighted sum of CO_2e for healthcare trips in the US in 2022	35 667 506 792 954 g CO_2e= 35.7 Mt CO_2e
Nationwide average amount of CO_2e emissions for one healthcare trip	10 172.3 g CO_2e/trip
Nationwide average amount of CO_2e emissions for healthcare trips per person per year	116.7 kg CO_2e/person/year
Nationwide average amount of miles traveled for healthcare trips per person per year	275 miles/person/year
Nationwide average amount of CO_2e emissions per mile traveled for healthcare visits[a]	424 g CO_2e/mile
Average amount of CO_2e emissions per mile traveled for healthcare visits for patients living in urban areas[b]	416 g CO_2e/mile
Average amount of CO_2e emissions per mile traveled for healthcare visits for patients living in rural areas[b]	445 g CO_2e/mile

Abbreviations: Mt= Megatons, CO_2e= Carbon dioxide equivalent. [a]To estimate the weighted nationwide average carbon emissions per mile traveled for healthcare visits in the US in 2022, we calculated the weighted total CO_2e emissions from all patient healthcare trips and divided this total by the weighted total miles traveled for healthcare visits in the same year. [b]Using the same methodology, we estimated the weighted average carbon emissions per mile traveled for healthcare visits separately for patients living in urban and rural areas.

© 2025 Zurl H et al. *JAMA Network Open*.

eTable 4. Weighted Number of Patients' Healthcare Trips by Fuel Type for Private Vehicles Used for Health Care Trips and by Sociodemographic Characteristics

Sociodemographic characteristic		Gas	Diesel	Plug-in hybrid	Electric	Non plug-in hybrid
**Overall**		2946295442 (93.3%)	18569528 (0.6%)	9308208 (0.3%)	53326200 (1.7%)	129204618 (4.1%)
**Urban/Rural**[a]	**Urban**	2306896544 (93.7%)	2910034.8 (0.1%)	9308208 (0.4%)	53326200 (2.2%)	89979270 (3.7%)
	**Rural**	639398898 (92.1%)	15659493 (2.3%)	0 (0%)	0 (0%)	39225349 (5.7%)
**Age**	**≤25**	117265843 (89.9%)	13211319 (10.1%)	0 (0%)	0 (0%)	0 (0%)
	**26-50**	1010751070 (94.4%)	0 (0%)	0 (0%)	8580346 (0.8%)	50992866 (4.8%)
	**51-75**	1555487296 (92.6%)	2910035 (0.2%)	9308208 (0.6%)	44745854 (2.7%)	67161513 (4.0%)
	**≥76**	262791232 (95.1%)	2448174 (0.9%)	0 (0%)	0 (0%)	11050239 (4.0%)
**Sex**	**Male**	1432443120 (94.4%)	18569528 (1.2%)	6031193 (0.4%)	44888394 (3.0%)	15109364 (1.0%)
	**Female**	1513852322 (92.3%)	0 (0%)	3277016 (0.2%)	8437806 (0.5%)	114095254 (7.0%)
**Race and Ethnicity**[b]	**NHW**	2061802946 (95.3%)	18569528 (0.9%)	9308208 (0.4%)	16225700 (0.8%)	56788906 (2.6%)
	**NHB**	279138290 (92.4%)	0 (0%)	0 (0%)	0 (0%)	23113600 (7.7%)
	**Hispanic**	529786608 (87.8%)	0 (0%)	0 (0%)	37100500 (6.2%)	36402912 (6.0%)
	**Other**[c]	75567599 (85.4%)	0 (0%)	0 (0%)	0 (0%)	12899200 (14.6%)
**Education**	**High School**	1196347286 (98.7%)	15659493 (1.3%)	0 (0%)	0 (0%)	0 (0%)
	**College/Bachelor**	1171801943 (87.6%)	2910035 (0.2%)	5859679 (0.4%)	50241099 (3.8%)	107575673 (8.0%)
	**Graduate**	578146213 (95.4%)	0 (0%)	3448530 (0.6%)	3085101 (0.5%)	21628945 (3.6%)
**Median Household Income in USD**	**≤ 25 000**	245142280 (98.0%)	2448174 (1.0%)	2582663 (1.0%)	0 (0%)	0 (0%)
	**25 000 – 49 999**	541867616 (96.9%)	0 (0%)	0 (0%)	0 (0%)	17310258 (3.1%)
	**50 000 - 999 999**	1058520695 (94.7%)	2910035 (0.3%)	3448530 (0.3%)	1626647 (0.1%)	50690450 (4.5%)
	**≥100 000**	1041530681 (88.9%)	13211319 (1.1%)	3277016 (0.3%)	51699554 (4.4%)	61203910 (5.2%)

© 2025 Zurl H et al. *JAMA Network Open*.

eTable 4. Weighted Number of Patients' Healthcare Trips by Fuel Type for Private Vehicles Used for Health Care Trips and by Sociodemographic Characteristics (continued)						
**Census Division**	**New England**	139253333 (89.8%)	0 (0%)	0 (0%)	0 (0%)	15837963 (10.2%)
	**Middle Atlantic**	401881172 (97.2%)	0 (0%)	3448530 (0.8%)	4547427 (1.1%)	3380277 (0.8%)
	**East North Central**	269517427 (10.0%)	0 (0%)	0 (0%)	0 (0%)	0 (0%)
	**West North Central**	264591133 (98.8%)	0 (0%)	0 (0%)	0 (0%)	3245580 (1.2%)
	**South Atlantic**	506618233 (84.0%)	13211319 (2.2%)	2582663 (0.4%)	8437806 (1.4%)	71954421 (11.9%)
	**East South Central**	239612868 (97.9%)	0 (0%)	3277016 (1.3%)	0 (0%)	1774498 (0.7%)
	**West South Central**	516513048 (99.7%)	0 (0%)	0 (0%)	0 (0%)	1693660 (0.3%)
	**Mountain**	298068368 (93.7%)	5358209 (1.7%)	0 (0%)	1613821 (0.5%)	12899200 (4.1%)
	**Pacific**	310239861 (84.4%)	0 (0%)	0 (0%)	38727147 (10.5%)	18419021 (5.0%)

Abbreviations: NHW= Non-Hispanic White, NHB= Non-Hispanic Black. [a]Urban/rural classification of participant households was determined using the U.S. Census Bureau's 2020 TIGER/Line Shapefile classification. [b]Race and ethnicity were self-reported by participants of the National Household Transportation Survey. [c]Other: Includes American Indian/Alaska Native, Asian, and Native Hawaiian/Pacific Islander.

© 2025 Zurl H et al. *JAMA Network Open*.

eFigure: Carbon Emissions per Healthcare Trip Across Different Patient Subgroups

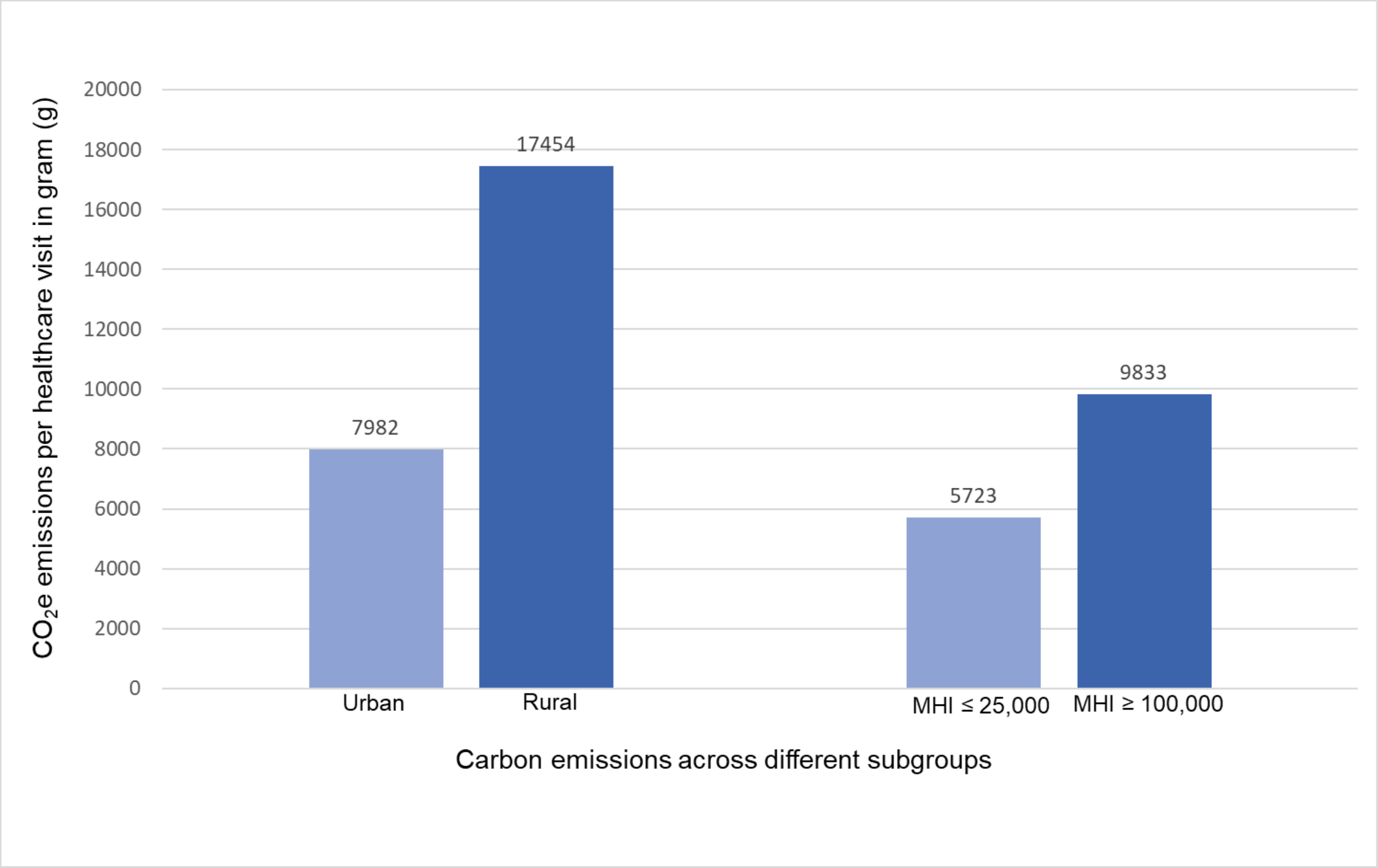

Abbreviation: MHI: Annual Median Household income in USD. Urban/rural classification of participant households was determined using the U.S. Census Bureau's 2020 TIGER/Line Shapefile classification.

© 2025 Zurl H et al. *JAMA Network Open*.
