# Supplementary material for: Carbon Emissions From Patient Travel for Health Care
Source: JAMA Netw Open. 2025 Mar 31;8(3):e252513. doi: 10.1001/jamanetworkopen.2025.2513 (PMC11959441; doi:10.1001/jamanetworkopen.2025.2513)
Supplement: Supplement 2. — Data Sharing Statement [file jamanetwopen-e252513-s002.pdf]

Data Sharing Statement

Zurl. Carbon Emissions From Patient Travel for Health Care. *JAMA Netw Open*. Published March 31, 2025. doi:10.1001/jamanetworkopen.2025.2513

Data
Data available: No

Additional Information
Explanation for why data not available: The original data is openly available online and can be obtained from the authors upon request
